# SUMOylation Is Required for Glycine-Induced Increases in AMPA Receptor Surface Expression (ChemLTP) in Hippocampal Neurons

**DOI:** 10.1371/journal.pone.0052345

**Published:** 2013-01-11

**Authors:** Nadia Jaafari, Filip A. Konopacki, Thomas F. Owen, Sriharsha Kantamneni, Philip Rubin, Tim J. Craig, Kevin A. Wilkinson, Jeremy M. Henley

**Affiliations:** School of Biochemistry, University of Bristol, Bristol, United Kingdom; University of Nebraska Medical Center, United States of America

## Abstract

Multiple pathways participate in the AMPA receptor trafficking that underlies long-term potentiation (LTP) of synaptic transmission. Here we demonstrate that protein SUMOylation is required for insertion of the GluA1 AMPAR subunit following transient glycine-evoked increase in AMPA receptor surface expression (ChemLTP) in dispersed neuronal cultures. ChemLTP increases co-localisation of SUMO-1 and the SUMO conjugating enzyme Ubc9 and with PSD95 consistent with the recruitment of SUMOylated proteins to dendritic spines. In addition, we show that ChemLTP increases dendritic levels of SUMO-1 and Ubc9 mRNA. Consistent with activity dependent translocation of these mRNAs to sites near synapses, levels of the mRNA binding and dendritic transport protein CPEB are also increased by ChemLTP. Importantly, reducing the extent of substrate protein SUMOylation by overexpressing the deSUMOylating enzyme SENP-1 or inhibiting SUMOylation by expressing dominant negative Ubc9 prevent the ChemLTP-induced increase in both AMPAR surface expression and dendritic SUMO-1 mRNA. Taken together these data demonstrate that SUMOylation of synaptic protein(s) involved in AMPA receptor trafficking is necessary for activity-dependent increases in AMPAR surface expression.

## Introduction

AMPA receptors (AMPARs) mediate most fast excitatory neurotransmission in the CNS and are key determinants of neuronal function and dysfunction [1], [2]. Increases in the number and changes in the composition and/or properties of synaptic AMPARs mediate long-term potentiation (LTP) of synaptic efficacy whereas their removal leads to long-term depression (LTD). AMPAR-mediated synaptic plasticity occurs at synapses throughout the CNS and underlies many neuronal, network and systems level functions ranging from sensory perception to intellectual processing [3].

AMPAR trafficking is complex and stringently regulated, and it is now clear that there are distinct pathways and multiple layers of control for the delivery, residence time and removal of synaptic AMPARs. The contribution of each pathway depends on specific signaling cues and the precise AMPAR subunit composition [1], [2]. Although remarkable progress has been achieved towards more complete understanding of the core mechanisms, fundamental questions remain about the function and dysfunction of AMPAR trafficking and synaptic plasticity in health and disease.

Posttranslational modification can direct protein folding, distribution, stability, activity and function. Consequently, posttranslational modifications are integral to signalling cascades, especially in the CNS where the processes affecting synaptic communication between neurons are highly orchestrated. For example both phosphorylation and ubiquitination have been extensively studied and play complex roles in postsynaptic protein regulation [4].

Protein modification by Small Ubiquitin-like MOdifier (SUMO) has emerged as an important regulator of neuronal function and dysfunction [5], [6]. SUMO is conjugated to lysine residues in target proteins by a three-enzyme pathway analogous to, but distinct from, ubiquinitation. A major difference is that unlike for ubiquitin where there are many E2 conjugating enzymes, Ubc9 is the only SUMO E2. There are 3 validated SUMO isoforms in mammals, designated SUMO-1-3. SUMO-2 and -3 differ by only 3 residues, and are collectively known as SUMO-2/3. SUMOylation is reversed via the actions of a family of SUMO-specific deconjugating enzymes, SENPs [7]. In general, SUMO acts by altering the protein interactions of the substrate protein. We have shown previously that SUMOylation of the kainate receptor subunit GluK2a at K886 is required for agonist-induced endocytosis of GluK2a-containing kainate receptors [8]. More recently, we have demonstrated that GluK2a SUMOylation is evoked by agonist-induced PKC phosphorylation of GluK2, and that this phospho-SUMOylation switch is a crucial determinant of kainate receptor LTD at mossy fibre synapses [9], [10], [11]. We, and others have also identified other synaptic SUMO substrates e.g. [12], [13] and SUMOylation has been implicated in several clinically important neurological and neurodegenerative diseases [14]. Interestingly, although there is currently no evidence to suggest that AMPARs are direct targets of SUMOylation [8], we have recently demonstrated that SUMOylation can regulate AMPAR trafficking during homeostatic synaptic plasticity [13].

We therefore investigated a potential role for SUMOylation in AMPAR surface expression mediated by more acute forms of plasticity using a well-characterised glycine stimulation protocol (ChemLTP) on cultured neurons [15], [16]. Our data show that ChemLTP stimulation recruited SUMO-1 to synapses and increased co-localisation of SUMO-1 with Ubc9. SUMO-1 and Ubc9 mRNAs were also increased and trafficked to dendrites following ChemLTP. Importantly, reducing global levels of protein SUMOylation by overexpression of a dominant negative Ubc9 or the catalytic domain of SENP-1 prevented the ChemLTP-induced increase in GluA1-containing AMPAR surface expression. These data indicate that SUMOylation of synaptic protein(s) involved in AMPAR trafficking is necessary for activity-dependent increases in AMPAR surface expression.

## Materials and Methods

### Primary neuronal culture

All experiments in this study were performed in accordance with UK Home Office Schedule 1 guidelines. Animals were sacrificed by cervical dislocation using procedures approved by the Home Office Licensing Team at the University of Bristol (UIN UB/12/008).

Rat embryonic hippocampal neuronal cultures were prepared from E18 Wistar rats using standard procedures. The culture medium was Neurobasal medium (Gibco) supplemented with B27 (Gibco) and 2 mM glutamine. For some experiments, neurons were transduced with Sindbis virus at DIV 17–20 and used 18–24 h later.

### Antibodies

The following antibodies are used: N-terminal anti-GluA1 (Millipore), anti PSD95 and anti- β-tubulin (Sigma), SUMO-1 and SUMO2 (hybridoma bank), UBC9, CPEB, MAP-2 (Sigma).

### Chemical LTP (Chem-LTP)

Chem-LTP was induced as described previously [15], [17], [18]. Briefly, neuronal cultures were transferred from Neurobasal growth medium to extracellular solution (ECS) containing: 150 mM NaCl, 2 mM CaCl_2_, 5 mM KCl, 10 mM HEPES (pH 7.4), 30 mM glucose, 0.5 µM TTX, 1 µM strychnine, 20 µM bicuculline methiodide. After 5 minutes in ECS, neuronal cultures were treated with glycine (200 µM) for 3 min in ECS then incubated in ECS without glycine for different time points (0, 5, 20, 40 min) prior to fixation.

### Immunocytochemistry

Following Chem-LTP induction neurons were fixed with 4% paraformaldehyde (PFA) in PBS for 5 min. Surface GluA1 was labelled with primary antibody against the N-terminal extracellular domain (Millipore) and a Cy3-conjugated secondary antibody (1∶500; Jackson Laboratories) under nonpermeant conditions. Neurons were then fixed for a further 12 min with 4% PFA, permeabilized using 0.1% Triton X-100 for 10 min and 10% horse serum and incubated in the same primary antibody and Cy5-conjugated secondary antibodies to visualise intracellular receptors. For simple immunolabeling of SUMO and Ubc9 or SUMO-1, after Chem-LTP induction, neurons were fixed and permeabilised with either 10 µg/ml digitonin 10 µg/ml or 0.1% Triton X-100 and 10% horse serum for 10 min. Cells were then stained with primary and secondary antibodies.

### Surface biotinylation

Following ChemLTP induction, neurons were chilled on ice, washed twice with ice-cold PBS and incubated with 0.15 mg/ml Sulfo-NHS-SS-Biotin (Pierce) in PBS for 10 min on ice. Cells were then washed two times with cold PBS, incubated for 5 minutes in 50 mM NH_4_Cl in PBS, washed three times with PBS and lysed in lysis buffer (150 mM NaCl, 25 mM HEPES, 1% Triton X-100, 0.1% SDS, 20 mM NEM). After centrifugation, lysate was incubated with streptavidin–agarose beads for 3 h at 4°C, washed four times in lysis buffer and bound proteins were detected by Western blotting.

### Fluorescence *in situ* hybridization

Staining was performed on cultured neurons plated on 13 mm glass coverslips as described previously using fluorescein-labeled cRNA probes [19]. Briefly, the following steps were performed: (i) fixation of tissue in 2% paraformaldehyde/2.5% sucrose for 20 min at room temperature (RT), (ii) permeabilization by dehydration and rehydration in graded ethanol solutions, (iii) prehybridization at room temperature, (iv) hybridization with full length CDS probes of rat SUMO-1, SUMO-2 or UBC9 overnight at 56°C, (v) posthybridization washes at 56°C with several changes of SSC buffer with increasing dilution at each rinse, (vi) probe detection, using peroxidase-conjugated anti-fluorescein antibody (Roche) followed by red (Cy3) fluorophore-based tyramide signal-amplification system (NEN, PerkinElmer). Subsequent immunofluorescent co-staining was performed for the dendritic marker MAP-2 (Sigma). Images were acquired as for immunocytochemistry and signal intensity was quantified with ImageJ.

### Images analysis and quantification

The images (collapsed stacks of no more than 3 optical sections) were taken using a Zeiss LSM 510 Meta confocal microscope 63× 1.32 NA objective, at 1024×1024 resolution. Images for all conditions in a particular experiment were obtained using identical acquisition parameters and were analyzed using ImageJ software (NIH). Neurons were selected blind based on EGFP fluorescence. Quantification of surface AMPARs was carried out by thresholding the EGFP or fluorescence signal in ImageJ to define outlines of neurons, and the ratio of surface over total (surface+internal) fluorescence within this area was calculated. These values were then normalized to the average surface fluorescence of untreated control cells. For *in situ* hybridization experiments images were analysed by an experimenter blinded to their identity. Outlines of dendrites and cell bodies were manually drawn in MAP-2 images and the masks were applied to the *in situ* hybridization image and average signal intensity was quantified. At least 10 cells (up to 5 ROI per cell) for each condition from at least three independent experiments were analysed.

For presentation, images were processed using Adobe Photoshop software (Adobe Systems) by adjusting brightness and contrast levels to the same degree for all conditions illustrated in each experiment. Colocalisation of different markers was determined using the colocalization plug-in in ImageJ. Values were then normalized to untreated control cells. At least 10 neurons and 3 to 5 ROIs for each condition from three independent experiments were analysed. Data are expressed as mean ± s.e.m and significance was determined using either one way ANOVA followed by Turkey's HSD post-hoc test or two-tailed *t*-tests, as indicated in the figure legends.

### Quantification of SDS–PAGE and immunoblots

Immunoblots from at least three independent experiments were scanned and analyzed using ImageJ.

### Data analysis and statistics

**Figure 1:**
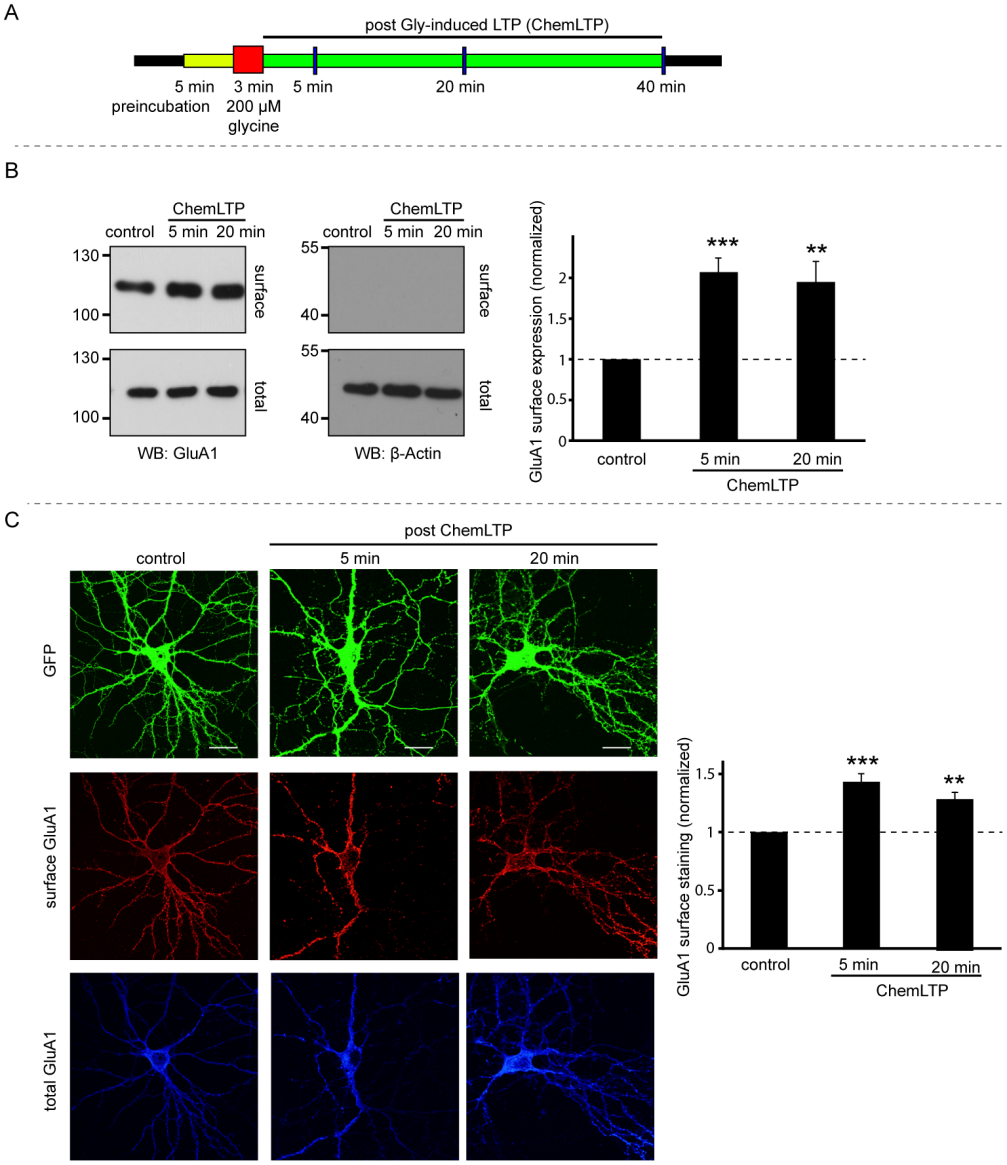
ChemLTP increases GluA1 surface expression. A) Schematic of the ChemLTP protocol. Dissociated hippocampal neuronal cultures (DIV 17–20) were treated with glycine (200 µM) for 3 min, then incubated in media without glycine for 5, 20 or 40 min. B) ChemLTP increases surface GluA1. Graph shows quantification of the ratio of surface to total GluA1. Statistical significance was determined using ANOVA. n = 5 ***p<0.005, ***p<0.001. C) ChemLTP Increases surface expression of endogenous GluA1-containing AMPARs in dissociated hippocampal neurons expressing EGFP. Surface and internal GluA1 were sequentially labeled under non-permeabilised and permeabilised conditions, respectively, with different coloured secondary antibodies. Representative images are shown. Graph shows surface:internal ratio relative to unstimulated control. n = 12–15 cells per condition in 3 independent experiments. ANOVA **p<0.005 and ***p<0.001. Scale bars – 20 µm.

**Figure 2:**
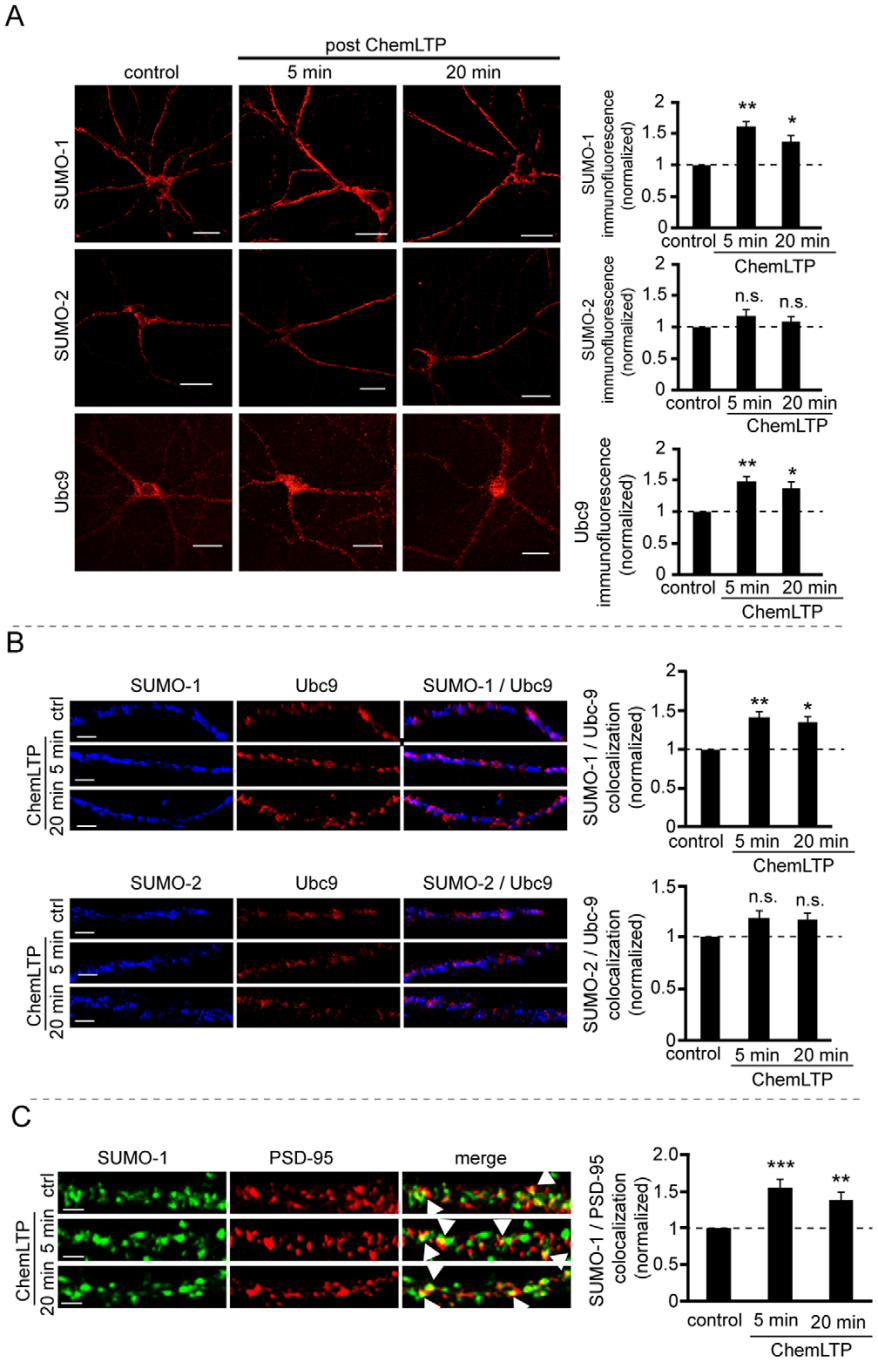
ChemLTP upregulates of SUMO-1 and Ubc9 protein levels. A) ChemLTP increases in SUMO-1 and Ubc9 immunoreactivity with no change in the intensity of SUMO-2 staining. Mean fluorescence intensity was analysed using ImageJ. Graphs show quantification of 3 independent experiments, 10 neurons per condition (ANOVA. *p<0.02; **p<0.005). Scale bars – 20 µm. B) ChemLTP enhances colocalization between SUMO-1 and Ubc9, and SUMO-2/3 and Ubc9 in cultured hippocampal neurons. Example of dendritic segments stained for SUMO-1 (upper panel) or SUMO-2 (lower panel) protein (blue) and Ubc9 (red). Colocalized puncta were quantified using JACoP plugin in ImageJ. Analysis was made in at least 3 different ROIs for each neuron (n = 10), for each condition in 3 independent experiments. (ANOVA. *p<0.02; **p<0.005). C) ChemLTP increases SUMO-1 clusters at synapses. Examples of fluorescence images of dendrites stained for SUMO-1 and PSD95. The colocalized clusters are indicated as yellow puncta. Graph shows quantification of SUMO-1/PSD95 colocalizing pixels. Analysis was performed in at least 3 independent experiments with 10 neurons per condition and 3 ROIs per neuron, using JACoP plugin in ImageJ. Statistical significance was determined using ANOVA. *p<0.02; **p<0.005.

**Figure 3:**
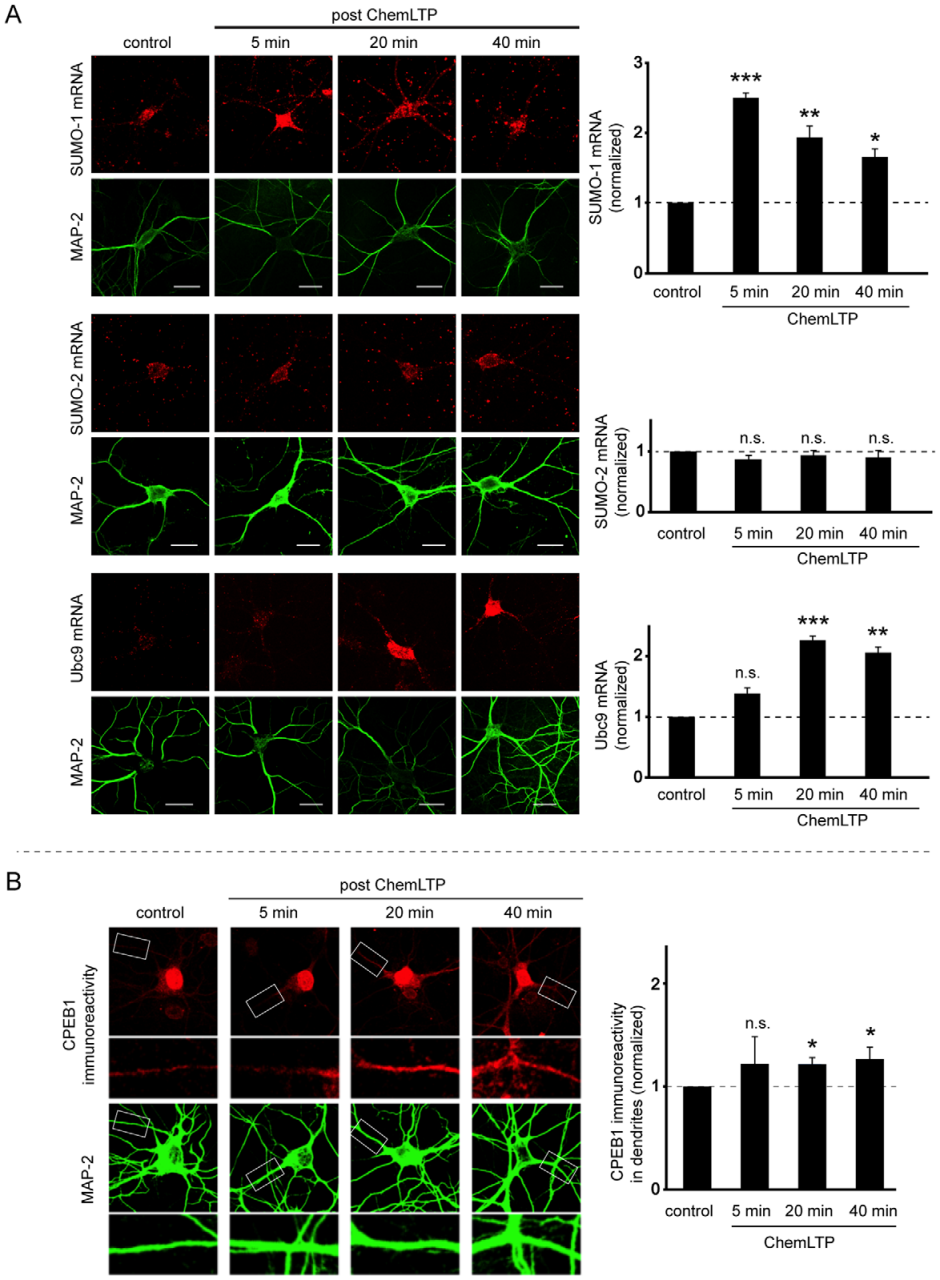
ChemLTP increases SUMO-1 and Ubc9 mRNA. A) Fluorescent in situ hybridization using cRNA probes for SUMO-1, SUMO-2 and Ubc9 (red) in hippocampal neurons co-stained with the dendritic marker MAP-2 (green). ChemLTP increased levels of SUMO-1 and Ubc9 but not SUMO-2 mRNA in soma and dendrites. Mean fluoresence intensity was quantified in at least 3 independent experiments with 10 neurons per condition and 3 ROIs per neuron using ImageJ. B) ChemLTP increases levels of the mRNA binding protein CPEB1 (red) in hippocampal cultured neurons co-immunostained with MAP2 (green). Three independent experiments with 8 neurons per condition and 4 ROIs per neuron were quantified using JACoP plugin in ImageJ. Statistical significance was determined using ANOVA *p<0.02; **p<0.005. Scale bars – 20 µm.

**Figure 4:**
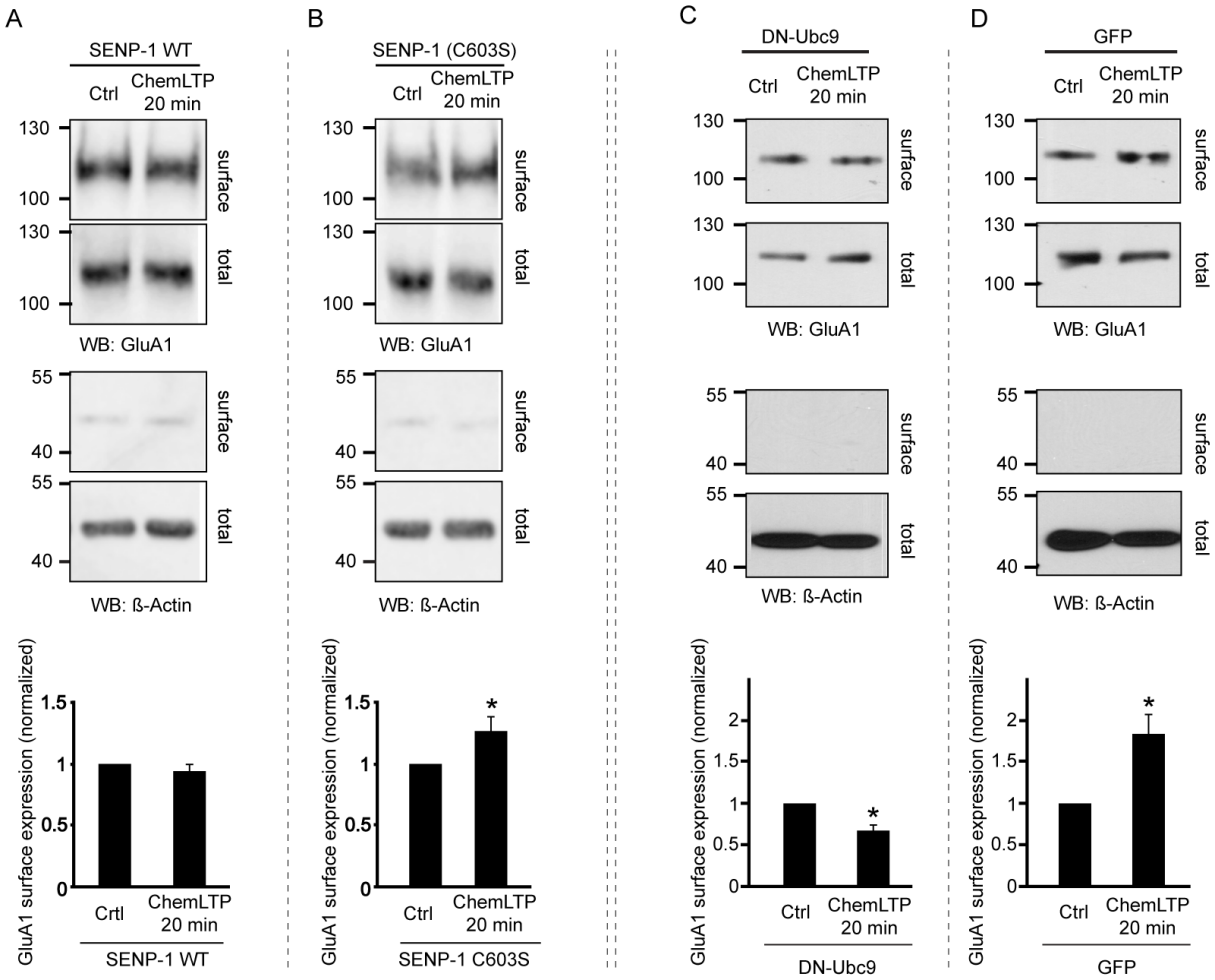
Overexpression of SENP-1 or dominant-negative Ubc9 inhibits the ChemLTP-mediated increase in surface GluA1. A, B) Overexpression of active SENP-1 (A) catalytic domain, but not inactive SENP-1(C603S) (B), abolishes the ChemLTP-mediated increase in surface expression of GluA1 as determined by surface biotinylation and immunoblotting. The corresponding bar graphs show quantification of surface/total GluA1 ratio. Statistical significance was determined using Student's t-test. C, D) Dominant-negative Ubc9 blocks the ChemLTP-mediated increase in surface GluA1. Neurons were infected with virus expressing dominant-negative Ubc9 (DN-Ubc9) (C) or control GFP (D) and subjected to ChemLTP. Surface biotinylation and western blotting were used to monitor changes in surface expression. Corresponding graphs show quantification of surface/total GluA1 ratio. Statistical significance was determined using Student's t-test. n = 4 (A and B) or n = 3 (C and D) *p<0.02.

**Figure 5:**
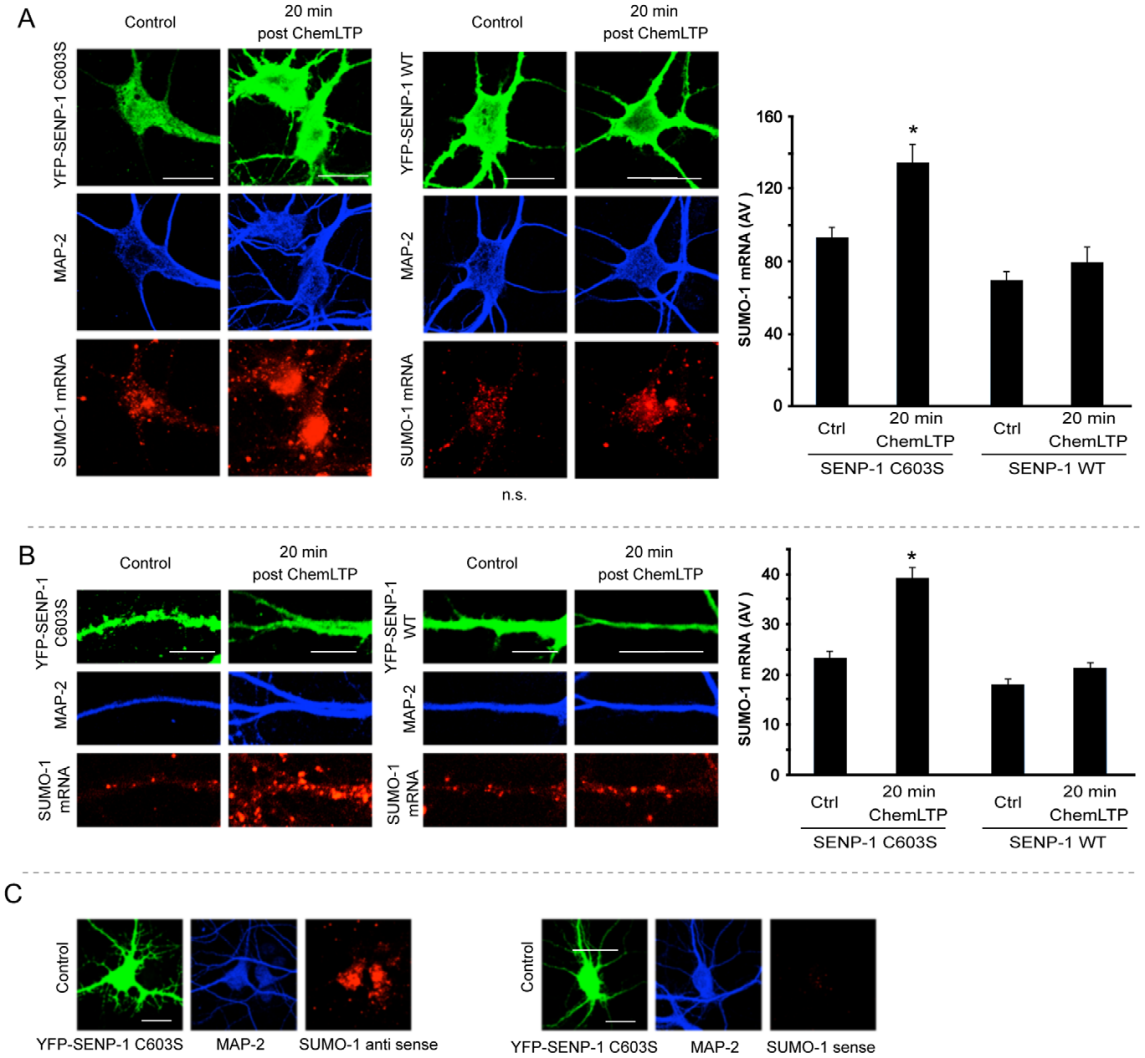
Overexpression of SENP-1 diminishes the ChemLTP-mediated increase in SUMO-1 mRNA. A) Representative images showing effects of ChemLTP in the soma and processes of hippocampal neurons overexpressing SENP-1 C603S (inactive control) or SENP-1 WT (green). Fluorescence *in situ* hybridization was performed using cRNA probes for SUMO-1 (red). Neurons were then co-stained with MAP-2 (blue). Graph shows quantification of mean fluorescence intensity (arbitrary units) in soma quantified from 4 independent experiments with 8–12 neurons per condition using ImageJ. B) Representative examples of dendritic regions of neurons overexpressing SENP-1 C603S (inactive control) or WT. Graph shows quantification of mean fluorescence intensity (arbitrary units) in dendrites. Quantification from 4 independent experiments; each experiment: 8–12 neurons per condition and 4 dendrites per neuron, done using ImageJ Scale bars – 5 µm. C) *In situ* hybridization with SUMO-1 negative control (sense probe) yielded virtually no signal compared to the anti-sense (experimental) probe. Scale bars – 20 µm. In all cases statistical significance was determined using ANOVA followed by Bonferroni post-hoc test (*p<0.01).

All quantitative data are presented as the means ± SEM. Significance values were determined using ANOVA for Figures 1, 2, and 3 (* <0.015, ** <0.005, *** <0.001). For Figure 4 the significance value is * <0.01. In Figure 5 only one experimental condition is compared to its control so significance was determined by t-test (* <0.05, ** <0.01, *** <0.001).

## Results and Discussion

### ChemLTP increases AMPAR surface expression

Bath application of 200 µM glycine for 3 min was used to activate synaptic NMDARs in conjunction with spontaneously released glutamate in dissociated cortical and hippocampal neuronal cultures (Figure 1A). The surface expression of AMPARs was assessed by surface biotinylation and immunostaining of GluA1. Surface GluA1 in cortical neurons increased to 207±16% after 5 minutes and 195±23% after 20 minutes post glycine stimulation (Figure 1B). Changes in localisation of native AMPAR subunits in hippocampal neurons were assessed using fixed cell immunocytochemistry. Surface GluA1 increased to 141±8% at 5 min and 130±7% 20 minutes after glycine application (Figure 1C). These findings are consistent with previous reports for ChemLTP [15], [17], [18], [20] and confirm that the protocol is effective and robust in our cultures.

### ChemLTP increases dendritic SUMO-1 and Ubc9

We next investigated the effects of ChemLTP on proteins involved in the SUMOylation pathway. We assessed the immunoreactivities for SUMO-1, SUMO2/3 and Ubc9 in dendritic regions of fixed, permeabilsed cultured hippocampal neurons (Figure 2A). ChemLTP caused a rapid increase in dendritic immunoreactivity of both SUMO-1 and Ubc9. Ubc9 staining was increased to 148±7% at 5 min and 137±9% at 20 min and SUMO-1 to 160±7% at 5 min and 136±8% at 20 min. Levels of SUMO-2/3 were unchanged. In addition to increasing dendritic levels of SUMO-1 and Ubc9, ChemLTP also increased their colocalisation in dendrites (140±6% at 5 min and 133±8% at 20 min following ChemLTP; Figure 2B). These data are consistent with priming of Ubc9 with SUMO-1 for covalent SUMOylation of target dendritic substrate proteins and/or direct SUMO-1-ylation of Ubc9 itself [21]. There were no significant changes in colocalisation between SUMO-2 and Ubc9 following stimulation.

We also determined if the increase in SUMO-1 occurs at, or close to postsynaptic sites by colocalisation of SUMO-1 and PSD95. Colocalisation between SUMO-1 and PSD95 was increased to 155±9% at 5 minutes and 139.6±8% at 20 minutes after ChemLTP compared to untreated controls (Figure 2C).

### ChemLTP increases SUMO-1 and Ubc9 mRNAs, and levels of the mRNA binding protein CPEB

We used fluorescence *in situ* hybridization (FisH) to monitor levels of SUMO-1, SUMO-2 and Ubc9 mRNAs. Neurons were stained with anti-MAP-2 antibody to visualize dendrites and mRNA levels were determined 5, 20 and 40 min after glycine stimulation. ChemLTP markedly increased SUMO-1 mRNA to 250±7% at 5 min; 195±15% at 20 min and 165±12% at 40 min compared to control levels. Similarly, mRNA for Ubc9 was increased to 138±9% at 5 min, 227±5% at 20 min and 207±8% at 40 min. As with SUMO-2 protein levels, SUMO-2 mRNA levels were unchanged by ChemLTP (Figure 3A).

Cytoplasmic polyadenylation element binding protein (CPEB) is an mRNA binding protein involved in translational repression in the nucleus but that can activate target RNA for translation [22] and increase dendritic transport of mRNAs, particularly those that have cytoplasmic polyadenylation element (CPE) [23]. Importantly, CPEB is involved in various forms of synaptic plasticity [24] including the maintenance of LTP [25], [26]. The 3′ UTR of rat SUMO-1 has 3 putative CPE sites upstream of two polyadenylation elements. ChemLTP caused a significant increase in dendritic CPEB immunoreactivity to 122±6% at 20 min and 126±10% at 40 min (Figure 3B).

### SENP-1 overexpression inhibits ChemLTP-induced increase in surface AMPARs

SENP-1 plays a role in SUMO maturation and cleaves covalently bound SUMO-1 and SUMO-2/3 from substrate proteins. Overexpression of the SENP-1 catalytic domain effectively deSUMOylates neuronal target proteins [27]. Our data indicate that ChemLTP recruits SUMOylation pathway proteins to dendrites and spines and increases colocalisation of SUMO-1 and Ubc9. We therefore asked whether protein SUMOylation is required for ChemLTP. In neurons overexpressing SENP-1 ChemLTP did not increase surface GluA1 (94±5% at 20 min after ChemLTP). As expected, however, ChemLTP effectively increased GluA1 surface expression in cells overexpressing the inactive point mutant SENP-1(C603S) (127±11% at 20 min; Figure 4A).

To further confirm the involvement of SUMOylation in ChemLTP we overexpressed a dominant-negative Ubc9 (DN-Ubc9; [28]). Neurons expressing GFP showed a robust increase in surface GluA1 in response to ChemLTP (182±22% at 20 min after ChemLTP), however this was blocked in neurons expressing GFP-DN-Ubc9 (Figure 4B). Indeed, unexpectedly, in cells expressing GFP-DN-Ubc9 we observed a significant decrease in surface GluA1 after ChemLTP (67±6% at 20 min after ChemLTP). While the reason for this decrease in GluA1 surface expression is unclear, taken together these data indicate that protein SUMOylation is required for the increase in GluA1 surface expression following ChemLTP.

### SENP-1 reduces the ChemLTP increased transcription of SUMO-1 mRNA

Our findings indicate that SUMO-1 is recruited to dendrites in response to ChemLTP. The mRNAs encoding many proteins involved in plasticity are translocated along dendrites and undergo local translation close to synapses. Having shown that protein deSUMOylation inhibits ChemLTP we next asked if deSUMOylation affected levels and dendritic delivery of SUMO-1 mRNA. We therefore tested the effects of SENP-1 overexpression on SUMO-1 mRNA expression and dendritic translocation. In control neurons expressing inactive SENP-1(C603S) there was a significant increase in the amount of SUMO-1 mRNA in the cell body (146±11%) at 20 minutes post ChemLTP. In neurons expressing active SENP-1, however, there was no significant change in SUMO-1 mRNA (Figure 5A). Similarly, in SENP-1(C603S) expressing neurons dendritic SUMO-1 mRNA significantly increased at 20 minutes after ChemLTP (168±10%), whereas there was no significant change in neurons expressing SENP-1 WT (Figure 5B). The specificity of the *in situ* hybridization procedure was validated using a sense probe, which yielded almost no signal compared to the anti-sense (experimental) probe (Figure 5C). These results suggest that the ChemLTP-induced increase SUMO-1 mRNA is mediated via a pathway that requires protein SUMOylation. Also, the rise in dendritic levels are consistent with local translation of SUMO-1 mRNA playing a role in glycine-induced long-term increase of surface AMPARs.

We show that SUMOylation is necessary for ChemLTP-induced increase of surface AMPARs. Blocking protein SUMOylation either through overexpression of the catalytic domain of SENP-1, which removes both SUMO-1 and SUMO-2/3 from substrates, or overexpresssion of a dominant negative form of Ubc9, the sole SUMO-specific conjugating enzyme, prevents the increase in surface AMPARs in response to glycine stimulation. These data strongly suggest that SUMOylation is required for the activity-dependent trafficking of AMPARs that underlies LTP.

We have previously demonstrated that protein SUMOylation regulates kainate receptor trafficking and plasticity [8], [9], [10].However, while the effect of protein SUMOylation on kainate receptor plasticity is mediated through direct modification of the KAR subunit GluK2 [10], we have been unable to detect direct SUMOylation of AMPAR subunits. Thus, SUMOylation of protein(s) involved in activity-dependent AMPAR trafficking is necessary for the ChemLTP-mediated increase in AMPAR surface expression. The identity of these proteins is currently unclear but there are multiple potential candidate SUMO substrate proteins that might directly or indirectly influence AMPAR trafficking [13], [29].

ChemLTP increases SUMO-1 and Ubc9 proteins in dendrites and an accumulation of SUMO-1 at synapses, visible as early as 5 minutes after glycine stimulation. In addition, ChemLTP leads to an increase in the colocalisation of SUMO-1 with Ubc9. This can represent either non-covalent SUMO loading of Ubc9 for subsequent conjugation to target proteins and/or direct SUMOylation of Ubc9 to regulate substrate discrimination [21]. Indeed, the latter possibility may provide a mechanism for targeting specific proteins for SUMOylation during LTP.

In addition to the recruitment and colocalisation of SUMO-1 and Ubc9, our fluorescence *in situ* hybridization data demonstrate that ChemLTP increases SUMO-1 and Ubc9 mRNA. Thus, ChemLTP stimulates both the transcription of SUMO-1 and Ubc9 mRNA and synaptic targeting of pre-existing SUMO-1 and Ubc9 proteins. Furthermore, our results suggest that SUMOylation of yet unknown target proteins regulates SUMO-1 mRNA transcription and possibly dendritic transport.

It is a well-established concept that translocation of mRNA to dendrites followed by local translation is an important mechanism contributing to LTP [30], [31]. ChemLTP increases SUMO-1 mRNA in the soma and dendrites suggesting ChemLTP-induced active transport of SUMO-1 mRNA to dendrites, further indicating that SUMO-1 is as a plasticity-related gene. Thus, the observed increase in SUMO-1 protein in dendrites at 5 minutes after stimulation may be due in part to local translation of pre-existing dendritic mRNA, which is subsequently replenished at later stages of LTP. Such mode of action has been observed in other plasticity-related mRNA species [19]. Consistent with this, ChemLTP increases the dendritic localization of the RNA binding protein CPEB-1. CPEB-1 mediates cytoplasmic activity-dependent mRNA polyadenylation and translation through binding to cytoplasmic polyadenylation elements (CPEs) with the consensus 5′-UUUUAU-3′ present in the 3′UTR of target mRNAs [24]. CPEB has also been shown to be important for the dendritic transport of mRNAs containing CPEs [23] but it has not yet been determined whether it interacts directly with SUMO-1 mRNA in neurons. However, taken together, these results support the concept that SUMO-1 is as a plasticity-related gene.

In summary, we show that SUMOylation is required for the initial insertion of AMPA receptors during the induction of ChemLTP. Additionally our data indicate that ChemLTP induction in turn upregulates the SUMOylation machinery at both the mRNA and protein levels, as well as increasing the association of SUMO-1 with the sole SUMO conjugating enzyme Ubc9.

Thus, we hypothesize that the initial AMPAR insertion observed upon the induction of LTP is mediated by either pre-existing SUMO-1 and Ubc9 proteins, or proteins newly translated from pre-existing dendritic mRNAs. Potentially, this initial increase is stabilized through the LTP-induced transcription of SUMO-1 and Ubc9 mRNAs and their further local translation in dendrites. While we do not yet know the identity of the SUMO substrate proteins that regulate these pathways there are many interacting and signaling proteins involved in AMPAR trafficking that either contain consensus SUMOylation motifs or have already been demonstrated to be SUMO targets [6]. Importantly, the function and dysfunction of neuronal SUMOylation has been strongly implicated multiple neurological and neurodegenerative diseases [14]. Thus, our findings highlight protein SUMOylation as a crucial mediator of AMPAR insertion during LTP and reveal a potential role for SUMOylation in the pathogenesis of disorders of the nervous system that are characterized by defective AMPAR trafficking and plasticity.
